# Preventive regimen of immunostimulants within personalised “3S” paradigm of anticancer drug testing

**DOI:** 10.1186/1878-5085-5-S1-A35

**Published:** 2014-02-11

**Authors:** Ekaterina Moiseeva

**Affiliations:** 1 Russian Academy of Science, Moscow, Russia

## 

Earlier, the personalised “3S” paradigm of preclinical anti-cancer drug testing was developed in parallel with a new conception of predictive, preventive, and personalised medicine. The paradigm includes three main points: 1) **S**et of complementary mouse models to reproduce a wide range of human breast cancer (BC) types; 2) **S**teps of preclinical anti-cancer drug testing procedure starting from *in vitro* and *in vivo* research on transplanted and spontaneous BC mouse models followed by probing of preventive regimen(s); (3) **S**tratified sampling as a methodology to distinguish more homogeneous subgroups within a heterogeneous population of experimental inbred mice using maximal number of individual recipient characteristics before and after testing procedure. This personalised procedure permits revealing of both positive and negative effects of a given drug.

Two types of different mouse models for preventive regimen testing exist within our paradigm; while commonly used preclinical drug testing procedures rarely include preventive regimen tests as one-level transplanted models are generally accepted. In contrast, our multistage conception proposes both transplanted and spontaneous BC mouse models using. The key advantage of a transplantable model is simultaneous developing of several homogenous populations of tumour-bearing mice. However, this mouse model preserves well-known limitations as healthy young recipient mice do not reflect numerous main characteristics of cancer patients that are mostly aged having a number of concomitant chronic inflammatory diseases. Furthermore, autochthonous carcinogenesis in humans and mice is a multi-staged process that is characterised as gradual transformation of cells and evolutionary expansion of cell populations *in situ*; and immune system is supposed to play a key role in this process. Earlier, we showed that our non-SPF conventional mouse strains with high frequency of spontaneous BC in aged females with preceded inflammatory diseases mimic the human BC aetiology/morphology properly. Several studies of preventive regimen tests in both transplanted and pre-cancerous spontaneous models are presented here to illustrate the acceptability of our paradigm. Firstly, a number of prestart transplanted mouse models of BC were used to test preventive regimens of immunotherapy by interleukin-2 and Imunofan^®^. In majority of our experiments, a single application of immunostimulants 3 days before tumour cell inoculation resulted in significant stimulation of tumour appearance in treated male recipients. In parallel, pre-cancerous BLRB and CBRB aged females were used as spontaneous models to test preventive regimens of the same immunostimulants. In contrast with above-mentioned data, clear age-related inhibition of BC appearance in pre-cancerous treated groups was observed. Transplanted mouse models hardly may be considered as an adequate model to test preventive regimens of immunotherapy against BC; but this test is suggested to compare extent of efficacy of various immunostimulants. While prophylactic anti-cancer effect of used drugs was revealed in aged pre-cancerous females, obtained contradictory data warn against uncontrolled usage of immunostimulants in breast cancer prone population. In summary, our original anti-cancer drug methodology demonstrates practical and scientific applicability.

